# Robust Rules for Optimal Colorimetric Sensing Based on Gold Nanoparticle Aggregation

**DOI:** 10.1021/acssensors.3c00287

**Published:** 2023-04-13

**Authors:** José Luis Montaño-Priede, María Sanromán-Iglesias, Nerea Zabala, Marek Grzelczak, Javier Aizpurua

**Affiliations:** † Department of Electricity and Electronics, FCT-ZTF, UPV-EHU, 48080 Bilbao, Spain; ‡ Donostia International Physics Center, Paseo Manuel de Lardizabal 4, 20018 Donostia-Sebastián, Spain; § Centro de Física de Materiales, (CSIC-UPV/EHU), Paseo Manuel de Lardizabal 5, 20018 Donostia-Sebastián, Spain

**Keywords:** colorimetric sensing, gold nanoparticles, geometrical parameters, clustering, numerical spectra, color difference, RGB color space, HSV color space

## Abstract

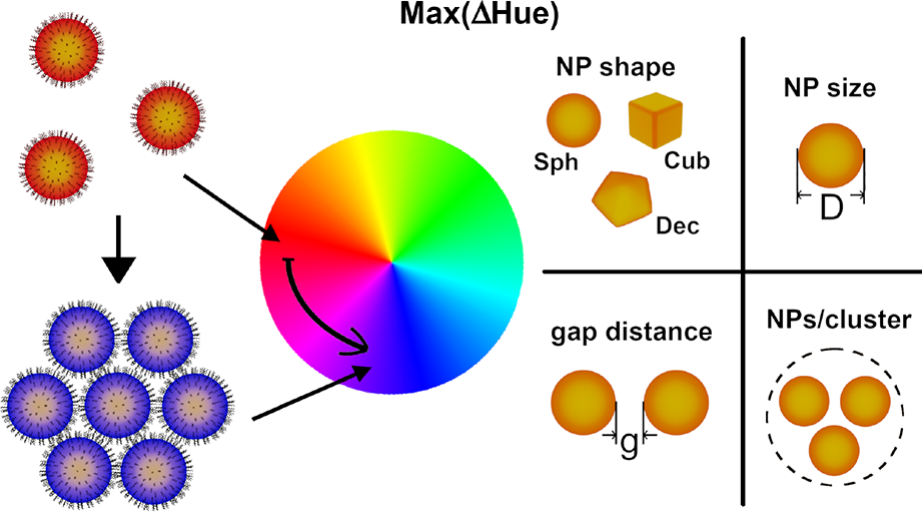

Spurred by outstanding optical properties, chemical stability, and facile bioconjugation, plasmonic metals have become the first-choice materials for optical signal transducers in biosensing. While the design rules for surface-based plasmonic sensors are well-established and commercialized, there is limited knowledge of the design of sensors based on nanoparticle aggregation. The reason is the lack of control over the interparticle distances, number of nanoparticles per cluster, or multiple mutual orientations during aggregation events, blurring the threshold between positive and negative readout. Here we identify the geometrical parameters (size, shape, and interparticle distance) that allow for maximizing the color difference upon nanoparticle clustering. Finding the optimal structural parameters will provide a fast and reliable means of readout, including unaided eye inspection or computer vision.

The working principle of biosensors built around plasmonic nanoparticles (NPs) is based on their localized surface plasmon resonances (LSPRs), coherent oscillations of the free electrons in metals usually driven by an incident electromagnetic wave.^1^ The most general approach for plasmonic sensing is based on a spectral shift of the plasmon resonance as a response to the selective binding of an analyte to a metal surface (change in local refractive index).^2−6^ Although highly reproducible and quantitative, plasmonic sensors based on spectral shifts are difficult to implement as a mass testing method since they usually require sophisticated optical readouts, such as spectrophotometers and data processing. Moreover, a single wavelength data analysis, often implemented in spectral sensors, can lead to information loss.^7^ Thus, spectral sensors are convenient for centralized laboratory testing, operated by expert personnel. On the other hand, the current pandemic has demonstrated that colorimetric sensing based on gold nanoparticles is a convenient tool for mass testing, where the untrained human eye completes the readout without medical assistance.^8−10^ Facile fabrication, low cost, fast readout, and societal acceptance pave the way toward further improvements of this type of sensor by taking advantage of the outstanding optical properties of gold nanoparticles.^11,12^

A convenient means of plasmonic sensing, particularly gold nanoparticles, involves their selective aggregation in the presence of surface-binding bio(macro)molecules. When a colloidal solution of gold plasmonic nanoparticles (e.g., spherical) aggregates in the presence of molecules (e.g., DNA), the solution turns from red to blue as a result of the so-called plasmon coupling.^13−16^ The readout assessment through color change does not require spectrophotometers and data interpretation by expert users. In addition, the readout can be digitalized in situ with mobile devices and converted into a given color space (RGB) that conveys more information than single-wavelength data analysis.^11,17−19^ Although sensing by aggregating nanoparticles has improved considerably over the last years, its commercial use remains out of reach. The main reason is that aggregating nanoparticles can lead to inhomogeneous interparticle distance and uncertainties in the number of nanoparticles per cluster or mutual arrangement affecting the readout consistency and reproducibility.

To tackle these issues, we establish a set of optimal parameters—size, shape, and interparticle distance of gold nanoparticles—that allow for reaching the largest possible color difference in visible spectral range upon clustering (Figure 1). As an experimental model implementation, we selected DNA-driven aggregation of nanoparticles stabilized with complementary single-stranded DNA. Numerical calculations and experimental data show that decahedra with an edge length of 30 nm forming clusters of 10 units at 2 nm interparticle distance are the best-performing nanoparticles in terms of red-to-blue color transition in RGB space.

**Figure 1:**
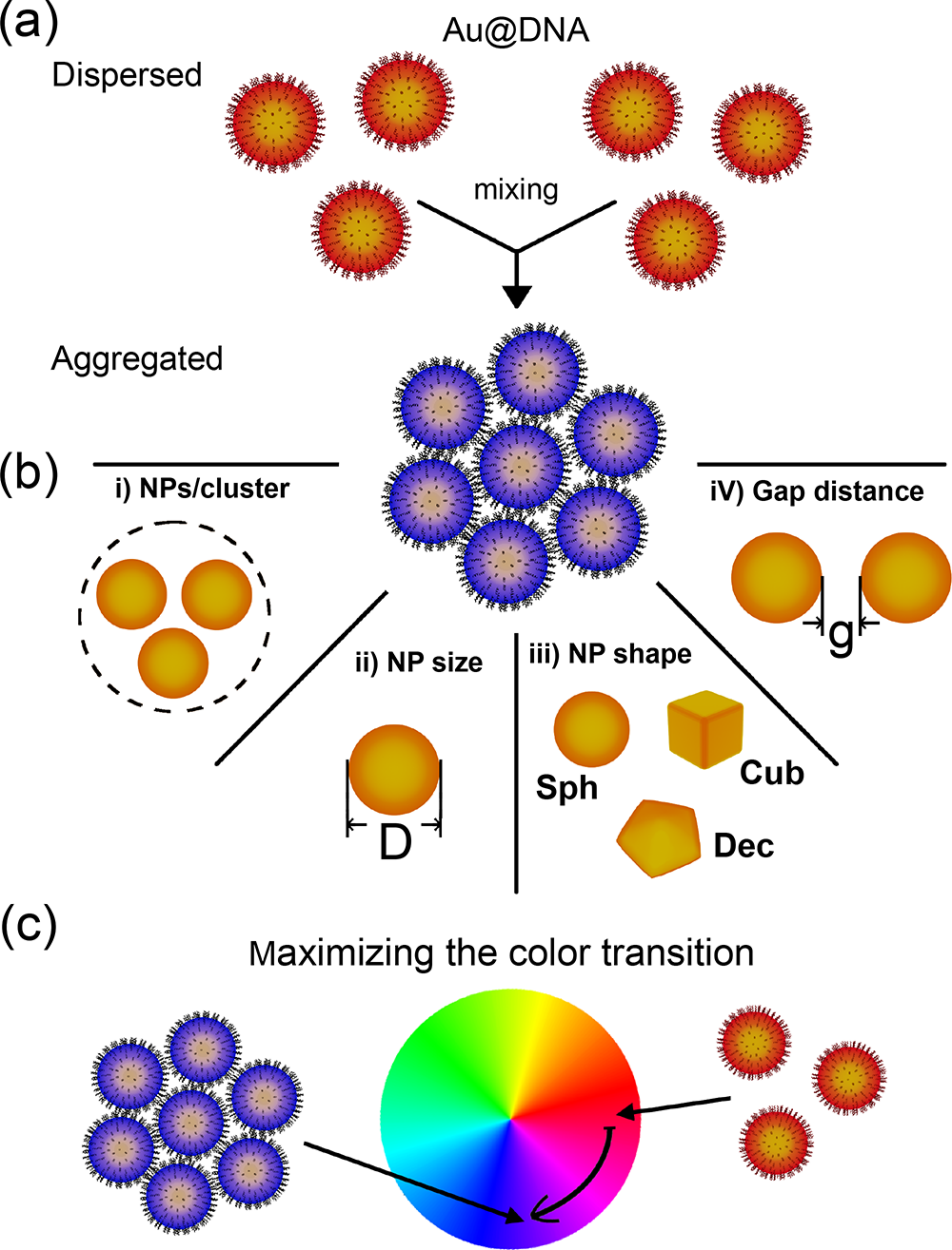
Geometrical parameters defining the performance of colorimetric sensing based on nanoparticle aggregation. (a) Selective binding of an analyte to the surface ligands induces aggregation of the initially stable plasmonic nanoparticles (gold), resulting in a gradual color transition from red to blue. (b) Geometrical parameters affecting the extent of color difference: (i) the number of NPs per formed cluster, (ii) diameter, (iii) shape, and (iv) gap between nanoparticles in the clusters. (c) Aggregation-induced color transition mapped on hue, saturation, value (HSV) or RGB color spaces.

## Results

A binary readout is often used for interpreting colorimetric biosensors (e.g., COVID-19 antigen and serological tests),^9,20,21^ in which red color indicates positive while no color indicates negative readout, for instance. In colorimetric testing based on the aggregation of nanoparticles, the readout is often binary as well. However, the limiting color is either red or blue, and the assignment to a negative or positive reading depends on the principle of operation of a given sensor.^14,15,22−24^ To ensure red-to-blue color transition upon aggregation, we selected spherical (Sph), cubic (Cub), and decahedral (Dec) nanoparticles that feature the primary LSPR in the visible spectral range and red-shifts upon clustering. Anisotropic gold nanoparticles such as rods, bipyramids, or plates^25^ were ruled out since they exhibit their primary LSPR in the infrared spectral range that can either be blue- or red-shifted depending on the mutual orientation. The selected size range was limited to the available experimental protocols for nanoparticles of reasonable monodispersity. Thus, for spherical nanoparticles, the diameter of choice ranged from 30 to 60 nm; for cubes, the side length was from 30 to 60 nm, and for decahedra, the edge length was from 20 to 50 nm (Figure S1, Figure S2, and Figure S3).

**Figure 2:**
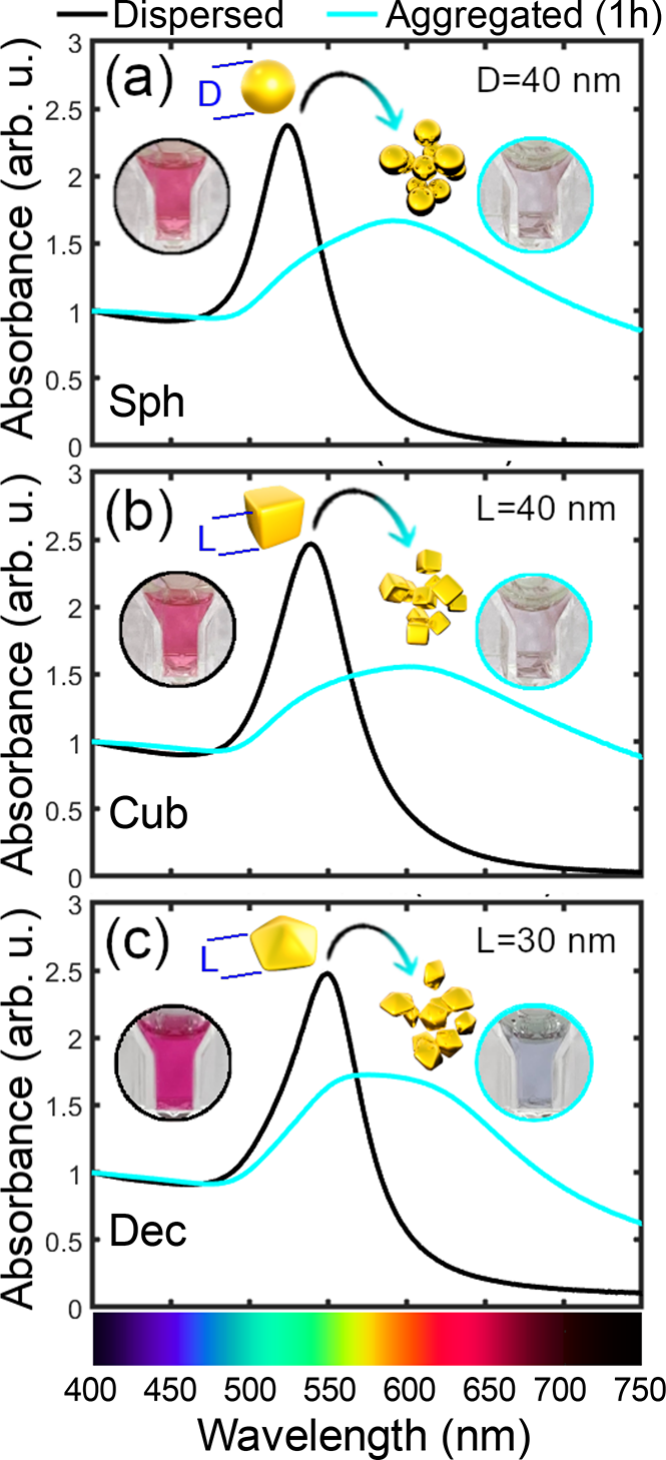
Typical spectral shift in colloidal sensors based on an aggregation of (a) spherical nanoparticles (Sph-NPs) of diameter *D*, (b) cubic nanoparticles (Cub-NPs) of length *L*, and (c) decahedral nanoparticles (Dec-NPs) of length *L*. The initially stable gold nanoparticles (black lines) undergo clustering (cyan lines) due to the hybridization of complementary DNA strands. The bars at the bottom represent the colors perceived by the human eye.^29^

To emulate a sensing event, we perform testing experiments on selected DNA-driven aggregation of gold nanoparticles functionalized with single-stranded DNA^26^ (Table S1). DNA is a versatile and tunable biomolecular platform driving nanoparticle aggregation, which has been proposed for numerous sensing applications, including food safety^27^ and liquid biopsy.^28^ When two batches of gold nanoparticles bearing complementary DNA are put in contact, aggregation starts, leading to a gradual color transition from red to blue (Figure 2). To study and quantify the sensing performance of the three selected types of NPs and obtain a comparable color estimation, we calculated the RGB values from both experimental and optical absorption spectra (see Section 1.3.3 in the Supporting Information). Note that, for the sake of simplicity, we used the Hue (*H*) parameter to classify color (Figure S6), which exhibits superior sensitivity to the environmental changes (e.g., aggregation).^11^

**Figure 3:**
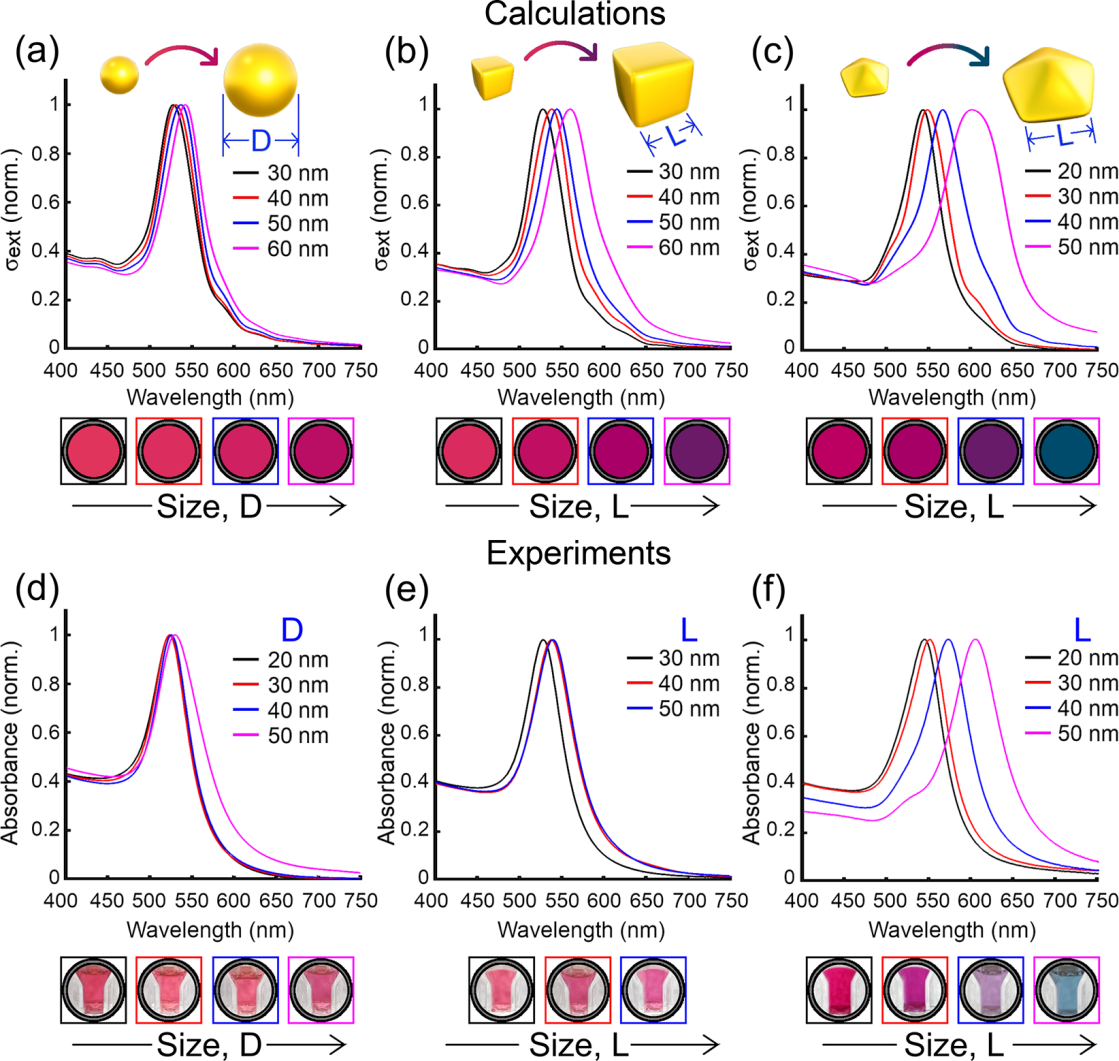
Calculated extinction cross-section spectra (σ_ext_, top panels) and experimental absorbance spectra (bottom panels) of (a, d) spherical, (b, e) cubic, and (c, f) decahedral single (dispersed) gold NPs. The RGB colors derived from σ_ext_ are shown at the bottom of the calculated spectra (a–c), and photos of the colloidal solutions are shown at the bottom of the absorbance spectra (d–f) for different sizes.

As mentioned above, the colorimetric sensor based on the aggregation of nanoparticles should operate between two limiting colors: red (dispersed nanoparticles absorbing in the blue-green range) and blue (aggregated nanoparticles absorbing in the yellow-red range). It is well-recognized that the absorption bands of dispersed nanoparticles red-shift with increasing size of nanoparticles, which can alter the initial red color. Our calculated (Figure 3a–c) and experimental (Figure 3d–f) spectra show that, indeed, as the size of the initial nanoparticles increases, the progressive red-shift of LSPR alters the red color. Furthermore, this effect is also dependent on the shape of the nanoparticles.

**Figure 4:**
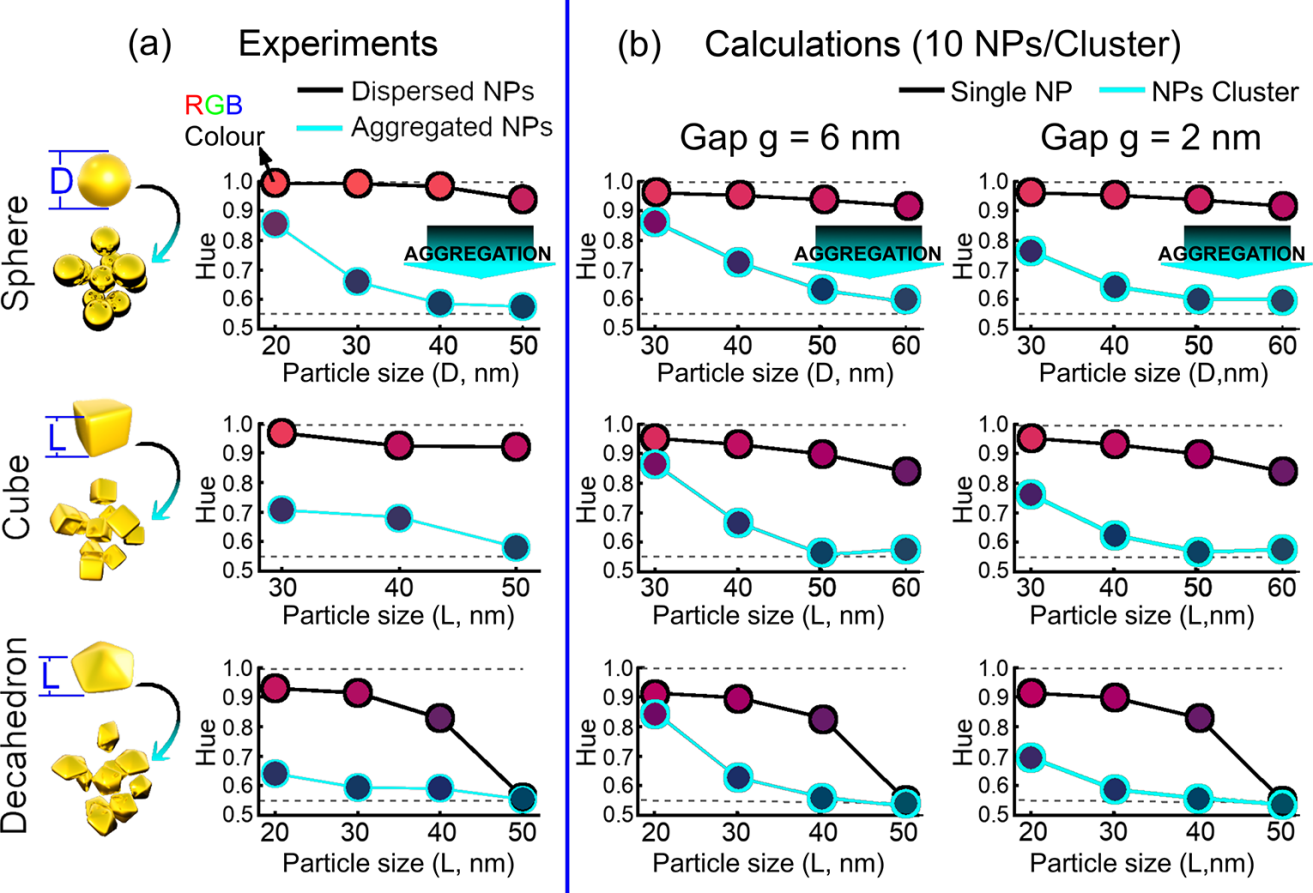
Color transition upon nanoparticle aggregation. (a) Experimental results show the change of color (through Hue values) from dispersed (black line) to aggregated (cyan line) for spherical, cubic, and decahedral NPs. (b) Theoretical results show the change of Hue values upon clustering of 10 NPs/cluster with an interparticle gap of (left column) 6 nm and (right column) 2 nm. The circle markers along the lines have the computed colors in RGB space obtained from spectral analysis.

The RGB color scheme can be summarized in the single-valued Hue number (*H*). For experimental dispersed Sph-NPs the Hue value remained invariant with increasing diameter from 20 nm (*H* = 0.99) to 50 nm (*H* = 0.94), thus conserving the red color of the solutions (Figure 4a, top panel). Dispersed Cub-NPs maintained a magenta color with increasing size but with a slight decrease in Hue value from 0.97 (30 nm) to 0.92 (50 nm) (Figure 4a, central panel). The color of the dispersions containing Dec-NPs turned from magenta (*H* = 0.93) to purple (*H* = 0.83) and to blue (*H* = 0.56) with increasing edge length from 20 and 40 to 50 nm, respectively (Figure 4a, bottom panel). The invariance of color with the increasing size of spherical nanoparticles suggests that this particular shape exhibits high tolerance to size polydispersity, which is quite the opposite behavior for faceted nanoparticles. The increase of electron interactions at more localized spaces in the Dec-NPs broadens the absorption spectrum,^30^ leading to a drastic change of the *H* value, which is more pronounced with increasing size distribution (Figure 4a, bottom panel). Thus, using faceted nanoparticles (e.g., cubes, or decahedra) in colorimetric sensing demands high monodispersity.

For dispersed colloids, the initial *H* value of 1 (red color) drops to 0.55 (blue color) during aggregation (see the Hue wheel in Figure S6 as reference). Note that *H* values that are between 0 and 0.55 (green and yellow colors, Figure S6) are inaccessible for aggregated nanoparticles since their absorption band falls precisely at the green and yellow spectral range (500–600 nm, cyan lines in Figure 2). Hence, the maximum allowed change of Hue values is 0.45 (1–0.55) for an aggregation-based colorimetric sensor (see the arrow in the Hue wheel, Figure S6). Our experimental data confirmed such a limit (Figure 4a). For the three shapes used, the *H* value is nearly unity while nanoparticles remain dispersed and progressively decreases toward the limiting value of 0.55 upon aggregation.

In a typical sensor based on nanoparticle aggregation, appreciable clustering occurs within the first few minutes after the addition of the analyte (Figure S8). The change of color during clustering stems from interparticle plasmon coupling that becomes dominant when the interparticle distance decreases and the number of particles per cluster increases.^31^ To evaluate the effect of interparticle distance on the color difference, we calculated the extinction spectra of clusters comprising 10 nanoparticles with interparticle gaps of 6 and 2 nm (Figure 4b, Figure S2). The RGB values generated from the extinction spectra confirmed that red-colored dispersed nanoparticles undergo a transition toward blue color with decreasing interparticle distance. At interparticle gap distance of 6 nm and small diameter, the calculated Hue values barely decreased (first purple point in the line of cyan points in Figure 4b), which is the experimental trend found for Sph-NPs, but not for Cub-NPs and Dec-NPs. In contrast, for larger dimensions, the theoretical Hue values decreased toward 0.55, corroborating the experimental trend in all cases. Further decrease of the interparticle gap distance down to 2 nm (keeping constant the number of 10 nanoparticles per cluster) showed an improved agreement of the Hue value between theory and experiments (blue points in Figure 4a,b), suggesting that this shorter distance reproduces more accurately the molecular dimensions in between the aggregated nanoparticles.

**Figure 5:**
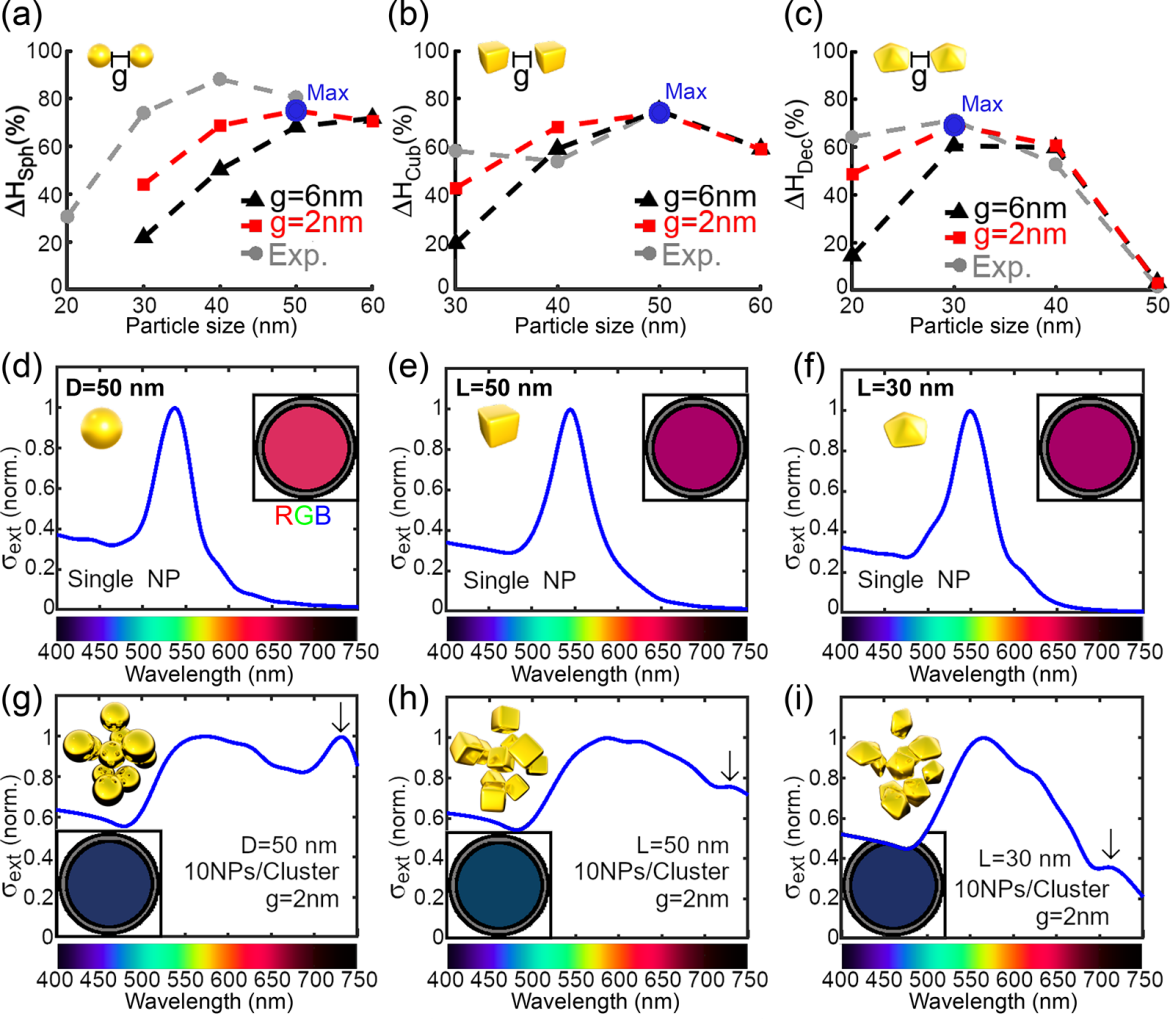
(a–c) FOM Δ*H* (eq 1) resulted from the theoretical and experimental Hue values (see Figure 4) of (a) spherical, (b) cubic, and (c) decahedral nanoparticle clusters (Δ*H*_Sph_, Δ*H*_Cub_, and Δ*H*_Dec_, respectively) of 10NPs/cluster with gap distances of *g* = 6 nm (theor., black triangle-marked lines), 2 nm (theor., red square-marked lines), and experimental Δ*H* (gray circle-marked lines). (d–i) Calculated extinction cross sections and RGB colors of (d, g) spherical, (e, h) cubic, and (f, i) decahedral single gold nanoparticles (d–f) and clusters (g–i), for the sizes in which Δ*H* is maximum for each theoretical case (see the blue circles in (a–c)). The bars at the bottom represent the colors perceived by the human eye.^29^

From the above analysis, one concludes that colorimetric sensing exhibits intrinsic limitations arising from two factors: (i) insufficient decrease of Hue value during aggregation, as observed experimentally for small nanoparticles, and (ii) small variation of Hue value of dispersed nanoparticles versus aggregated ones (experimentally and theoretically) for large nanoparticles, particularly decahedra with 50 nm of edge length. To quantitatively account for these two aspects we develop the following Figure of Merit (FOM) as a general descriptor for colorimetric sensing, namely, the relative change of Hue value upon aggregation
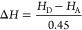
1where *H*_D_ and *H*_A_ are the Hue values of dispersed and aggregated states, respectively, normalized by the maximum possible change. Thus, the larger the Δ*H*, the greater the colorimetric sensitivity. By taking the calculated Hue values from both experimental and theoretical results in Figure 4, we calculated Δ*H* as a function of the nanoparticle size for the three types of particles (i.e., Δ*H*_Sph_, Δ*H*_Cub_, and Δ*H*_Dec_, respectively, in Figure 5a–c). As expected, the efficiency of colorimetric sensing based on the red-to-blue color transition showed a peak centered at an optimal (maximum) diameter window for each shape, where the values of Δ*H* are the highest (∼80%). On the left side of the peak, Δ*H* is rather small because the plasmon coupling is not pronounced enough (even at a 2 nm gap) due to the small volume of nanoparticles. On the right side of the peak, the values of Δ*H* decreased because the dispersed nanoparticles are large enough to partially overlap the spectral range corresponding to red. As a result, the large nanoparticles in dispersed mode exhibit blue color that, upon aggregation, does not undergo further transition, hence, the smaller Δ*H* in this size range. These results show that the optimal dimensions (the highest Δ*H*) are spheres and cubes of 50 nm and decahedra of 30 nm.

The theoretical predictions for maximum Δ*H* align well with the values obtained experimentally (dashed lines in Figure 5a–c). In particular, this is valid for cubes and decahedra (Figure 5b,c). Decahera of 30 nm received the highest values of Δ*H* equal to 72% and 70% for experimental and calculated spectra, respectively (Figure 5c). Similarly, for cubelike nanoparticles, the maximum theoretical value obtained was Δ*H* = 75% at an edge length of 50 nm, as observed experimentally (Figure 5b). The spherical maximum values of Δ*H* showed slight discrepancies between experimental and theoretical data. The experimental value was 88% for 40 nm nanoparticles (Figure S9), while the theoretical was 75% for 50 nm (Figure 5a). We postulate that such a discrepancy in diameter between theory and experimental values is due to (1) anisotropy considered in the theoretical clusters altering absorption bands at longer wavelengths (Figure 5g, arrow) and (2) heterogeneous interparticle distance in experimental samples (sub-2 nm) altering the plasmon coupling and thus shifting the maximum of Δ*H* to lower particle diameters.

As a general trend, one can notice that, for small diameters (20–30 nm), the theoretical values of Δ*H* are smaller than those observed experimentally (Figure 5a–c). This discrepancy stems from the fact that, in our calculations, we limited the number of NPs per cluster to 10 while in colloidal solution the cluster grew in size until phase separation.

Next, we analyze the calculated extinction cross-section spectra and RGB color transitions of the largest color difference (Figure 5d–i), showing that the important optimization procedure does not rely on the starting color of the dispersed solution but rather on the color difference after aggregation. For example, by turning blue upon aggregation, the red-colored spherical NPs of *D* = 50 nm allow for a similar colorimetric difference (Figure 5d,g) as the initially magenta-colored decahedral NPs of *L* = 30 nm, also turning blue upon aggregation (Figure 5f,i). This behavior is corroborated by the experimental results obtained for the same particle dimensions (Figure S9). Note that the small bands emerging at longer wavelengths in the spectra of aggregated nanoparticles are caused by the longitudinal modes of NP chains sustained in the clusters^32,33^ (indicated by black arrows in Figure 5g–i). Such bands further red-shift with increasing nanoparticles and cluster size (more nanoparticles per cluster). Besides, the wide band at lower wavelength stems from the contributions of the plasmon absorption of individual NPs and the interactive dimers in the cluster, which slightly redshift with the NP and cluster sizes.^32,33^

One can wonder which parameter—the interparticle distance or the number of particles per cluster—has the largest impact on the colorimetric sensitivity. To answer this question, we estimated the Δ*H* values for model clusters containing 6 and 10 nanoparticles and interparticle distances of 12, 6, and 2 nm (Figure S11). For decahedra, Δ*H* increased four times upon decreasing the interparticle distance from 12 to 6 nm at 6 NPs/cluster. Further 3-fold increase in Δ*H* was observed while decreasing the gap from 6 to 2 nm (10 NPs/cluster) for a decahedron of 20 nm. Interestingly, the effect of interparticle distance on the color difference was more pronounced for smaller diameters (20–40 nm). The values of Δ*H* were barely affected for larger diameters (e.g., 50 nm). Overall, these results suggest that the interparticle gap, rather than the size of the clusters, has a major effect on the colorimetric transition for nanoparticles, given that nanoparticles are of small diameter.^34^

**Figure 6:**
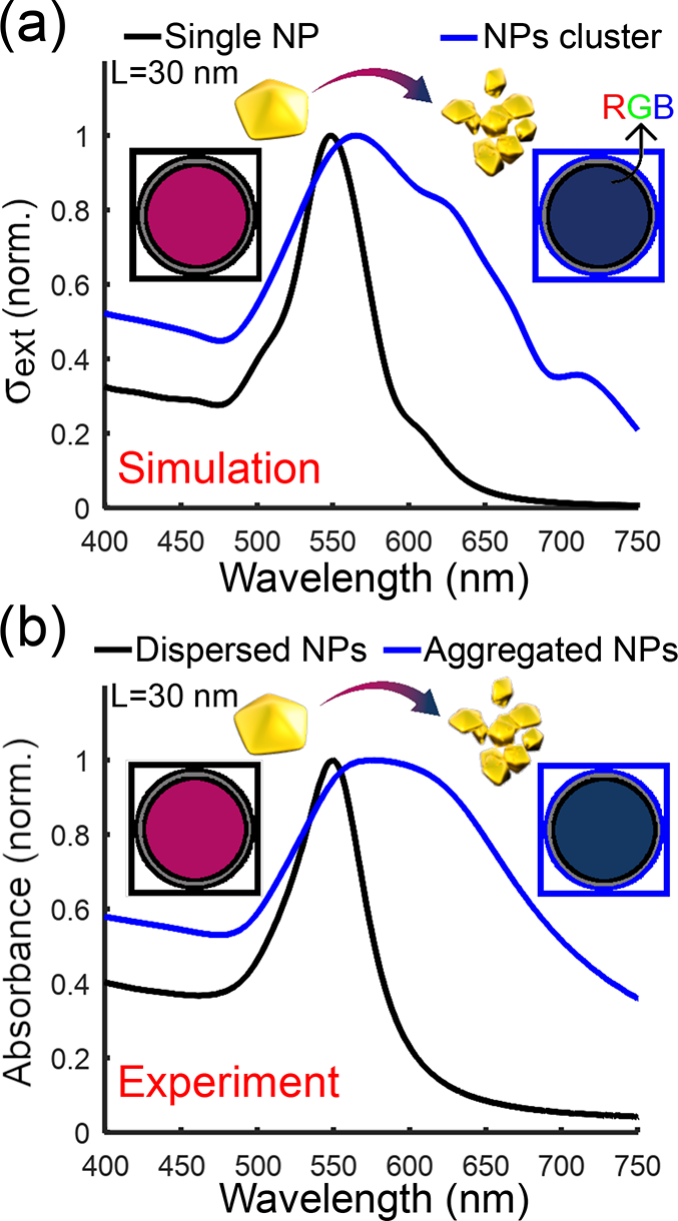
Optical properties of optimal nanoparticle shape – decahedra −. (a) Calculated extinction cross-section spectra of decahedral Au NPs with edge size *L* = 30 nm: single NP (black) and a cluster of 10-NPs with 2 nm-gap (blue). (b) Experimental absorbance spectra of a sample containing decahedra nanoparticles. (insets) Calculated RGB colors corresponding to each situation.

In light of the potential application in sensing, our results suggest that gold decahedra of 30 nm are the optimal shapes for efficient colorimetric sensors. Although Δ*H* for spheres is larger by 17% than that of decahedra, the volume of the decahedra (15 909 nm^3^ for an edge length of 30 nm) is four times smaller than the volume of the sphere (65 460 nm^3^ for a diameter of 50 nm), which reduces the amount of noble metal needed for sensing purposes, making decahedra a preferable choice. Figure 6 shows the theoretical extinction cross-section and experimental absorbance spectra for gold decahedra with an edge length of 30 nm. The experimental and theoretical spectral shifts and RGB color transitions correlate very well for 10 nanoparticles per cluster at a 2 nm interparticle gap, suggesting that the color transition can already be appreciable for the human eye for relatively small cluster sizes.^35^

To put the proposed FOM here in a general context of quantifying the difference between two colors, we compared all Δ*H* values with that of a standard measure, namely, Δ*E*_76_^36,37^ (Section 4 in Supporting Information). Δ*E*_76_ is a numerical scale quantifying the color difference in the CIE 1976 L*a*b* color space. We found that all nanoparticle shapes and cluster configurations studied here fall in the range of 3.5 < Δ*E*_76_ < 5, indicating that the aggregation of nanoparticles can be clearly differentiated and noticed by an inexperienced observer (Table S2). However, we found that the Δ*H* indicator is a more convenient measure for our purpose than Δ*E*_76_, since we aimed at maximizing the difference between two colors (upper bound) rather than defining a lower color contrast threshold.

## Discussion

In summary, our experimental and theoretical results of the absorbance during particle aggregation are put in the context of binary colorimetric sensing, where the transition from red to blue indicates the presence or absence of an analyte under study that induces NPs aggregation. By comparing experimental and theoretical data, we found that gold decahedrons of 30 nm in edge length are the best-performing nanoparticle morphology for colorimetric sensing (red-to-blue) based on aggregation. The analysis presented here focused on aggregating one type of nanoparticles, i.e., of the same size and shape. The aggregation of nanoparticles via biomacromolecules, in particular, DNA, makes heterogeneous clustering possible, where one can use two batches of nanoparticles of different shapes. Such a strategy can lead to the formation of satellitelike structures with much richer optical responses as compared to homogeneous clusters of the same shape. The systematic codification of specific cluster architectures with their optical outcome at the aggregated state can open up the possibility of colorimetric multiplexing. The proposed methodology of calculating Hue values (and the FOM Δ*H*) from input spectra opens up the possibility for tracking color transition in real-time through computer vision, where the resolution between colors of such transitions can be considerably augmented. As such, finding a linear relationship between the Hue value transition and the analyte concentration would pave the way for a quantitative readout.

## Supporting Information Available

Experimental and Theoretical methods. Figure S1: TEM images. Figure S2: Estimated gap between particles. Figure S3: Fitted curves to experimental absorbance spectra. Figure S4: Color matching functions. Figure S5: Standard iluminant D65. Figure S6: Hue wheel. Figure S7: Experimental absorbance spectra of dispersed and aggregated NPs. Figure S8: Aggregation kinetics of NPs. Figure S9: Change of Hue value for experimental samples. Figure S10: Calculated extinction cross sections of single NPs and NPs clusters. Figure S11: Change of Hue values for the calculated cases. Table S1: DNA sequences used. Table S2: Theoretical and experimental color differences (Δ*E*_76_) (PDF)

## References

[ref1] Soler M.; Lechuga L. M. Principles, technologies, and applications of plasmonic biosensors. J. Appl. Phys. 2021, 129, 111102 10.1063/5.0042811.

[ref2] Anker J. N.; Hall W. P.; Lyandres O.; Shah N. C.; Zhao J.; Van Duyne R. P. Biosensing with plasmonic nanosensors. Nat. Mater. 2008, 7, 442–453. 10.1038/nmat2162. 18497851

[ref3] Li M.; Cushing S. K.; Wu N. Plasmon-enhanced optical sensors: a review. Analyst 2015, 140, 386–406. 10.1039/C4AN01079E. 25365823 PMC4274271

[ref4] Tang L.; Li J. Plasmon-based colorimetric nanosensors for ultrasensitive molecular diagnostics. ACS Sens 2017, 2, 857–875. 10.1021/acssensors.7b00282. 28750528

[ref5] Chen Y.; Ming H. Review of surface plasmon resonance and localized surface plasmon resonance sensor. Photonic Sens 2012, 2, 37–49. 10.1007/s13320-011-0051-2.

[ref6] He M.-Q.; Yu Y.-L.; Wang J.-H. Biomolecule-tailored assembly and morphology of gold nanoparticles for LSPR applications. Nano Today 2020, 35, 101005 10.1016/j.nantod.2020.101005.

[ref7] Otte M. A.; Sepúlveda B.; Ni W.; Juste J. P.; Liz-Marzán L. M.; Lechuga L. M. Identification of the optimal spectral region for plasmonic and nanoplasmonic sensing. ACS Nano 2010, 4, 349–357. 10.1021/nn901024e. 19947647

[ref8] Alafeef M.; Pan D. Diagnostic approaches for COVID-19: lessons learned and the path forward. ACS Nano 2022, 16, 11545–11576. 10.1021/acsnano.2c01697. 35921264

[ref9] Huang C.; Wen T.; Shi F.-J.; Zeng X.-Y.; Jiao Y.-J. Rapid detection of IgM antibodies against the SARS-CoV-2 virus via colloidal gold nanoparticle-based lateral-flow assay. ACS Omega 2020, 5, 12550–12556. 10.1021/acsomega.0c01554. 32542208 PMC7241732

[ref10] Moitra P.; Alafeef M.; Dighe K.; Frieman M. B.; Pan D. Selective naked-eye detection of SARS-CoV-2 mediated by N gene targeted antisense oligonucleotide capped plasmonic nanoparticles. ACS Nano 2020, 14, 7617–7627. 10.1021/acsnano.0c03822. 32437124

[ref11] Reinhard I.; Miller K.; Diepenheim G.; Cantrell K.; Hall W. P. Nanoparticle design rules for colorimetric plasmonic sensors. ACS Appl. Nano Mater. 2020, 3, 4342–4350. 10.1021/acsanm.0c00475.

[ref12] Ventura B. D.; Cennamo M.; Minopoli A.; Campanile R.; Censi S. B.; Terracciano D.; Portella G.; Velotta R. Colorimetric test for fast detection of SARS-CoV-2 in nasal and throat swabs. ACS Sens 2020, 5, 3043–3048. 10.1021/acssensors.0c01742. 32989986 PMC7534800

[ref13] Zeng J.; Zhang Y.; Zeng T.; Aleisa R.; Qiu Z.; Chen Y.; Huang J.; Wang D.; Yan Z.; Yin Y. Anisotropic plasmonic nanostructures for colorimetric sensing. Nano Today 2020, 32, 100855 10.1016/j.nantod.2020.100855.

[ref14] Yu W.; Zhang T.; Ma M.; Chen C.; Liang X.; Wen K.; Wang Z.; Shen J. Highly sensitive visual detection of amantadine residues in poultry at the ppb level: A colorimetric immunoassay based on a Fenton reaction and gold nanoparticles aggregation. Anal. Chim. Acta 2018, 1027, 130–136. 10.1016/j.aca.2018.04.035. 29866262

[ref15] Xianyu Y.; Wang Z.; Jiang X. A plasmonic nanosensor for immunoassay via enzyme-triggered click chemistry. ACS Nano 2014, 8, 12741–12747. 10.1021/nn505857g. 25423357

[ref16] Zou L.; Shen R.; Ling L.; Li G. Sensitive DNA detection by polymerase chain reaction with gold nanoparticles. Anal. Chim. Acta 2018, 1038, 105–111. 10.1016/j.aca.2018.07.006. 30278890

[ref17] Guner H.; Ozgur E.; Kokturk G.; Celik M.; Esen E.; Topal A. E.; Ayas S.; Uludag Y.; Elbuken C.; Dana A. A smartphone based surface plasmon resonance imaging (SPRi) platform for on-site biodetection. Sens. Actuators B: Chem. 2017, 239, 571–577. 10.1016/j.snb.2016.08.061.

[ref18] Dutta S.; Saikia K.; Nath P. Smartphone based LSPR sensing platform for bio-conjugation detection and quantification. RSC Adv. 2016, 6, 21871–21880. 10.1039/C6RA01113F.

[ref19] Berg B.; Cortazar B.; Tseng D.; Ozkan H.; Feng S.; Wei Q.; Chan R. Y.-L.; Burbano J.; Farooqui Q.; Lewinski M.; Di Carlo D.; Garner O. B.; Ozcan A. Cellphone-based hand-held microplate reader for point-of-care testing of enzyme-linked immunosorbent assays. ACS Nano 2015, 9, 7857–7866. 10.1021/acsnano.5b03203. 26159546

[ref20] Guo Y.; Zhou Y.; Xiong S.; Zeng L.; Huang X.; Leng Y.; Xiong Y. Natural enzyme-free colorimetric immunoassay for human chorionic gonadotropin detection based on the Ag^+^-triggered catalytic activity of cetyltrimethylammonium bromide-coated gold nanoparticles. Sens. Actuators B Chem. 2020, 305, 127439 10.1016/j.snb.2019.127439.

[ref21] Jiang K.; Wu J.; Qiu Y.; Go Y. Y.; Ban K.; Park H. J.; Lee J.-H. Plasmonic colorimetric PCR for Rapid molecular diagnostic assays. Sens. Actuators B Chem. 2021, 337, 129762 10.1016/j.snb.2021.129762.

[ref22] Iglesias M. S.; Grzelczak M. Using gold nanoparticles to detect single-nucleotide polymorphisms: toward liquid biopsy. Beilstein J. Nanotechnol. 2020, 11, 263–284. 10.3762/bjnano.11.20. 32082965 PMC7006498

[ref23] Nie X.-M.; Huang R.; Dong C.-X.; Tang L.-J.; Gui R.; Jiang J.-H. Plasmonic ELISA for the ultrasensitive detection of Treponema pallidum. Biosens. Bioelectron 2014, 58, 314–319. 10.1016/j.bios.2014.03.007. 24662060

[ref24] de la Rica R.; Stevens M. M. Plasmonic ELISA for the ultrasensitive detection of disease biomarkers with the naked eye. Nat. Nanotechnol. 2012, 7, 821–824. 10.1038/nnano.2012.186. 23103935

[ref25] Sánchez-Iglesias A.; Winckelmans N.; Altantzis T.; Bals S.; Grzelczak M.; Liz-Marzán L. M. High-yield seeded growth of monodisperse pentatwinned gold nanoparticles through thermally induced seed twinning. J. Am. Chem. Soc. 2017, 139, 107–110. 10.1021/jacs.6b12143. 28009166

[ref26] Sanromán-Iglesias M.; Garrido V.; Gil-Ramírez Y.; Aizpurua J.; Grzelczak M.; Grilló M.-J. Plasmon-assisted fast colorimetric detection of bacterial nucleases in food samples. Sens. Actuators B: Chem. 2021, 349, 130780 10.1016/j.snb.2021.130780.

[ref27] Garcia Gonzalez J.; Hernandez F. J. Nuclease activity: an exploitable biomarker in bacterial infections. Expert Review of Molecular Diagnostics 2022, 22, 265–294. 10.1080/14737159.2022.2049249. 35240900

[ref28] Li J.; Li Y.; Pan L.; Pan W.; Li N.; Tang B. Spherical nucleic acids-based biosensors for cancer biomarkers detection. TrAC 2022, 157, 116807 10.1016/j.trac.2022.116807.

[ref29] Mather J. Spectral and XYZ Color Functions. 2023; https://www.mathworks.com/matlabcentral/fileexchange/7021-spectral-and-xyz-color-functions.

[ref30] Montaño Priede J. L.; Pal U. Estimating near electric field of polyhedral gold nanoparticles for plasmon-enhanced spectroscopies. J. Phys. Chem. C 2019, 123, 11833–11839. 10.1021/acs.jpcc.9b01105.

[ref31] Kelesidis G. A.; Gao D.; Starsich F. H.; Pratsinis S. E. Light Extinction by Agglomerates of Gold Nanoparticles: A Plasmon Ruler for Sub-10 nm Interparticle Distances. Anal. Chem. 2022, 94, 5310–5316. 10.1021/acs.analchem.1c05145. 35312292 PMC8988125

[ref32] Taylor R. W.; Esteban R.; Mahajan S.; Aizpurua J.; Baumberg J. J. Optimizing SERS from gold nanoparticle clusters: Addressing the near field by an embedded chain plasmon model. J. Phys. Chem. C 2016, 120, 10512–10522. 10.1021/acs.jpcc.6b00506.

[ref33] Esteban R.; Taylor R. W.; Baumberg J. J.; Aizpurua J. How chain plasmons govern the optical response in strongly interacting self-assembled metallic clusters of nanoparticles. Langmuir 2012, 28, 8881–8890. 10.1021/la300198r. 22364608

[ref34] Park S. Y.; Lee J.-S.; Georganopoulou D.; Mirkin C. A.; Schatz G. C. Structures of DNA-linked nanoparticle aggregates. J. Phys. Chem. B 2006, 110, 12673–12681. 10.1021/jp062212+. 16800601

[ref35] Sánchez-Iglesias A.; Claes N.; Solís D. M.; Taboada J. M.; Bals S.; Liz-Marzán L. M.; Grzelczak M. Reversible clustering of gold nanoparticles under confinement. Ang. Chem. Int. Ed. 2018, 57, 3183–3186. 10.1002/anie.201800736. PMC6468316 29417726

[ref36] González-Alcalde A. K.; Reyes-Coronado A. Large angle-independent structural colors based on all-dielectric random metasurfaces. Opt. Commun. 2020, 475, 126289 10.1016/j.optcom.2020.126289.

[ref37] Mokrzycki W. S.; Tatol M. Colour Difference Δ*E* - a Survey. Mach. Graph. Vis. 2011, 20, 383–411.

