# Supplementary material for: Robust Rules for Optimal Colorimetric Sensing Based on Gold Nanoparticle Aggregation
Source: ACS Sens. 2023 Apr 13;8(4):1827–34. doi: 10.1021/acssensors.3c00287 (PMC10152487; doi:10.1021/acssensors.3c00287)
Supplement: Supplementary file 1 — se3c00287_si_001.pdf [file se3c00287_si_001.pdf]

Robust Rules for Optimal Colorimetric Sensing Based on Gold Nanoparticle Aggregation Supporting Information

José Luis Montaño-Priede,[†,‡] María Sanromán-Iglesias,[¶] Nerea Zabala,[†,‡,¶] Marek Grzelczak,[*,¶,‡] and Javier Aizpurua[*,¶,‡]

†*Department of Electricity and Electronics, FCT-ZTF, UPV-EHU, Bilbao, 48080, Spain*

‡*Donostia International Physics Center (DIPC), Paseo Manuel de Lardizabal 4, 20018 Donostia-Sebastián, Spain*

¶*Centro de Física de Materiales (CSIC-UPV/EHU), Paseo Manuel de Lardizabal 5, 20018 Donostia-Sebastián, Spain*

E-mail: marek.g@csic.es; aizpurua@ehu.eus

Contents

1 Methods **3**

1.1 Materials . . . 3

1.2 Synthesis and functionalization of gold nanoparticles . . . 3

1.3 Theoretical methods . . . 5

1.3.1 Geometrical considerations . . . 5

1.3.2 Extinction cross section calculation . . . 7

1.3.3 Conversion of optical spectra into RGB and HSV color models . . . 8

2 Optical characterization of experimental samples **13**

3 Optical characterization of theoretical clusters **16**

4 Comparative between ΔE_{76} and ΔH **18**

References **20**

1 Methods

1.1 Materials

Gold (III) chloride trihydrate ($HAuCl_4 \cdot 3\,H_2O$), sodium borohydride ($NaBH_4$), cetyltrimethylammonium bromide (CTAB), ascorbic acid (AA), cetyltrimethylammonium chloride (CTAC, 25 wt.% in water), benzyldimethylhexadecylammonium chloride (BDAC), sodium hypochlorite, sodium bromide (NaBr), sodium dodecyl sulphate (SDS) 98%, sodium chloride (NaCl) 99.5%, and phosphate buffer (PB) 1 M, pH 7.4 were purchased from Sigma-Aldrich and used without further purification. Milli-Q water was used in all experiments. Thiolated oligonucleotides were purchased from Biomers (Germany).

1.2 Synthesis and functionalization of gold nanoparticles

Gold nanospheres.[1] Gold nanoparticles were obtained through two-step overgrowth process. Typically, initial seeds were prepared by reduction of $HAuCl_4$ (5 mL, 0.25 mM) with a strong reducing agent, $NaBH_4$ (0.3 mL, 10 mM) in aqueous CTAB solution (100 mM). To ensure complete decomposition of the reducing agent, the mixture was left undisturbed at 27 °C for 0.5 h. An aliquot of as-prepared seed (0.11 mL) was added to a growth solution containing CTAC (20 mL, 200 mM), $HAuCl_4$ (20 mL, 0.5 mM) and AA (15 mL, 100 mM). The mixture was left undisturbed at 27 °C for 30 min. To remove remaining reagents, the solution was centrifuged (1 h, 14000 rpm) and redispersed in water. The gold nanospheres (∼10 nm) of the resulting solution were used as seeds in the second overgrowth step to obtain 20 nm, 30 nm, 40 nm, and 50 nm nanoparticle sizes. In doing so, a solution of gold nanospheres (0.285 mL, 0.18 mL, 0.08 mL, and 0.01 mL at 3 mM for spheres of 20 nm, 30 nm, 40 nm, and 50 nm in diameter, respectively) was added to an aqueous growth solution of BDAC (50 mL, 100 mM), $HAuCl_4$ (0.25 mL, 50 mM) and AA (0.25 mL, 100 mM) at 40 °C and vigorous stirring. The mixture was left undisturbed at 30 °C for 30 min. To remove excess of reagents, the solution was centrifuged twice (6500 rpm, 30 min) and redispersed in

water.

Gold nanocubes.[1] To three solutions containing CTAC (5 mL, 15 mM) and Au seeds of 20 nm (0.61 mL, 0.46 mL, and 0.24 mL, 7.5 mM, for cube nanoaprticles of 30 nm, 40 nm, and 50 nm in length, respectively) were added NaBr (0.5 mL, 10 mM), AA (0.0188 mL, 100 mM) and $HAuCl_4$ (0.025 mL, 50 mM). The mixtures were left under stirring for 30 min at room temperature.

Gold decahedra.[2] Gold decahedra were prepared through seeded-growth methods. First, to prepare gold seeds, a freshly prepared $NaBH_4$ solution (0.25 mL, 25 mM) was added to a CTAC solution (10 mL, 50 mM) containing $HAuCl_4$ (0.25 mM) and citric acid (5 mM) under vigorous stirring at room temperature. After 2 minutes, the vial was closed and the seed solution was heated in an oil bath at 80 °C for 90 minutes. Then, to prepare gold decahedra, a solution of gold seeds (4 mL, 0.65 mL, 0.2 mL, and 0.1 mL for decahedral nanoparticles of 20 nm, 30 nm, 40 nm, and 50 nm in edge length) was added under vigorous stirring to a growth solution containing BDAC (100 mL, 100 mM), $HAuCl_4$ (1 mL, 50 mM) and AA (0.75 mL, 100 mM) at 30 °C. The mixture was left undisturbed at 30 °C for 30 minutes.

Ligand exchange.[3] Prior to the functionalization of gold nanoparticles with DNA, the as-prepared nanoparticles were subjected to ligand exchange to replace native surfactant molecules with citrate ions.

DNA functionalization.[4] Citrate-stabilized nanoparticles were functionalized with thiolated oligonucleotides (see Table S1). A solution of gold nanoparticles (1 mL, 0.5 M of Au) was mixed with SDS (100 μL, 0.01%), PB (10 μL, 1 M) and thiolated oligonucleotides (20 µL, 100 µM). The mixture was incubated at room temperature for 20 min. Next, a salt aging process was carried out where a solution containing NaCl (2 M), SDS (0.01%), and PB (0.01 M) was added sequentially to the mixture containing nanoparticles and oligonucleotides, in the following aliquots: 5 μL, 5 μL, 15 μL, 25 μL, and 50 μL, reaching a final NaCl concentration of 0.2 M. Each salt aging step was alternated with sonication (10 s) and incubation

(20 min), followed by incubation for 12 h. To remove excess oligonucleotides, the solutions were centrifuged (decahedra: 3000 rpm, 30 min, 2 times; nanocubes: 3500 rpm, 45 min, 2 times; nanospheres: 6000 rpm, 30 min, 2 times) and redispersed in SDS (0.01%). The final concentration of nanoparticles was 1 mM in terms of Au atoms for all samples.

Table S1: DNA sequences used to stabilize nanoparticles where spacer18 refers to polyethylene-glycol.

Name	Oligonucleotide Sequence
1Duplex	5´SH-Spacer18-AAC-GAC-TCA-TAT-TAA-3´
2Duplex	5´SH-Spacer18-TTA-ATA-TGA-GTC-GTT-3´

DNA-driven aggregation. Equal volumes (10 μL, 1 mM) of two batches of DNA-functionalized gold nanoparticles (Au@1Duplex and Au@2Duplex) were combined in a cuvette, followed by the addition of NaCl (8 μL, 5 M). To this solution was added 72 µL of water to reach the final volume of 100 μL. The mixture was left undisturbed at 25 °C for 30 min and characterized by UV-Vis-NIR.

1.3 Theoretical methods

1.3.1 Geometrical considerations

Upon analyte addition to the solution of nanoparticles, aggregation commences to produce clusters containing several nanoparticles accompanied by the change of color.[5–8] To study the effect of geometrical parameters (shape, size, and interparticle distance of nanoparticles) on color transition, we simulated the optical response of clusters comprising 6 and 10 nanoparticles. The following considerations were taken into account to set the upper and lower limits of the gap between nanoparticles in the aggregated state. The radially distributed DNA strands on the surface of the nanoparticles remain hydrated in a buffer solution to form a brush of approximately 11 nm.[9] Thus, to set the upper limit of a 12 nm gap, we assumed that DNA strands of adjacent nanoparticles intercalate, forming a double-stranded DNA bridge. The lower limit of 2 nm was set by estimating the mean interparticle distance (2.77

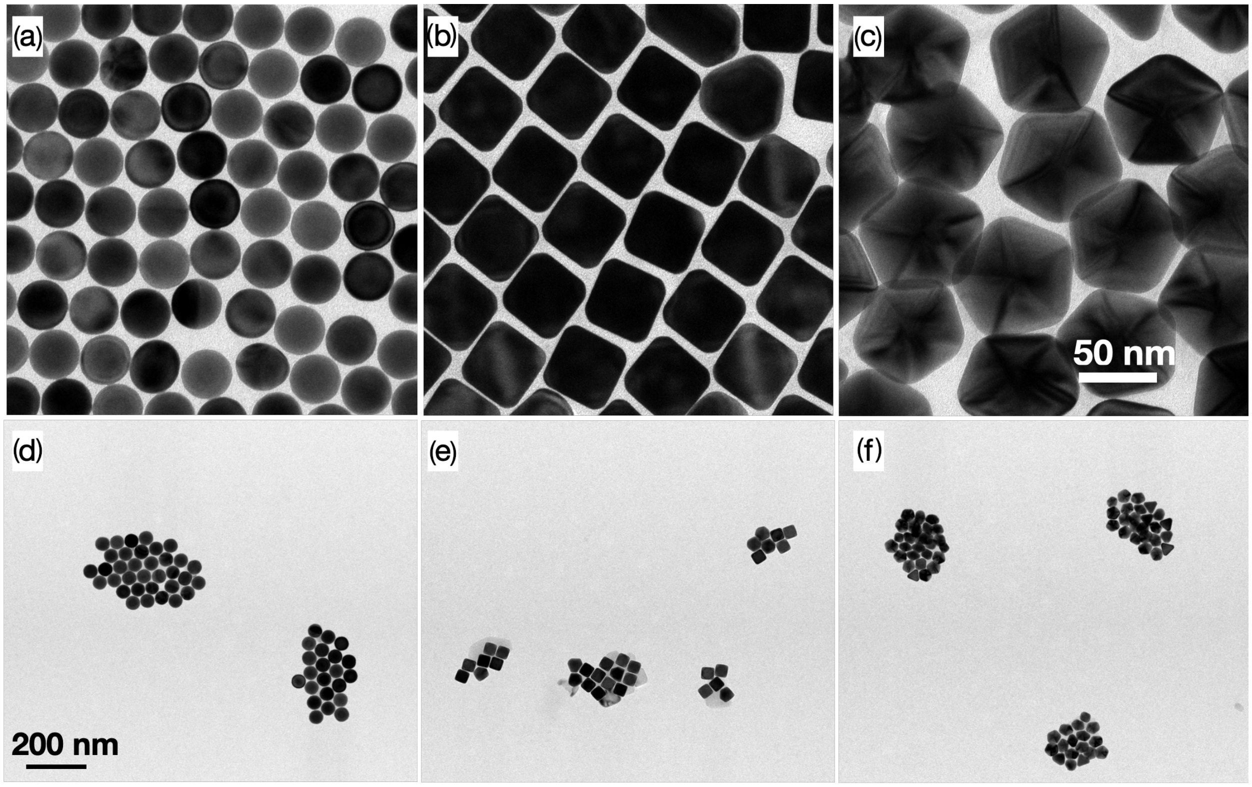

Figure S1: Representative TEM micrographs of selected gold nanoparticles.(a-c) TEM micrographs of selected gold nanoparticles. a) Spherical nanoparticles of 30 nm in diameter, b) cube-like nanoparticles of 30 nm in edge length, and c) decahedra nanoparticles with 40 nm in edge length. (d-f) Low-resolution TEM images of aggregated nanoparticles of the corresponding shapes.

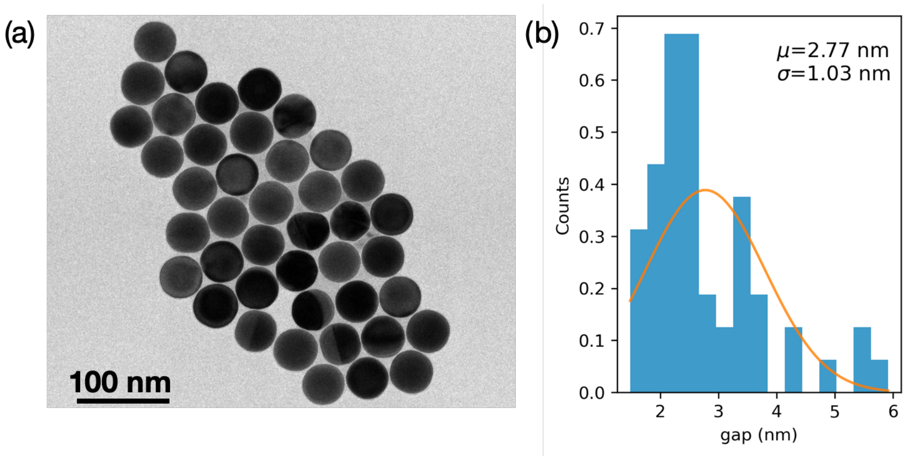

Figure S2: a) Representative TEM micrograph of aggregated spherical gold nanoparticles. b) Distribution of interparticle distances.

± 1.03 nm) from transmission electron microscopy (TEM) analysis of aggregated spherical nanoparticles (Figure S2). It should be stressed that TEM imaging imposes vacuum conditions where the organic shell is fully dehydrated and collapsed, making a 2 nm gap during aggregation in the liquid phase a less probable scenario in our experimental model. Nevertheless, such a small interparticle distance remains relevant for other molecular analytes (*e.g.*, heavy metal ions), motivating us to keep a 2 nm of lower limit of the interparticle gap in our calculations. Finally, the cluster shape was considered as isotropic and of compact structure. Recent studies have shown that clustering of DNA-functionalized nanoparticles follows reaction-limited aggregation implying nearly spherical shape.[10,11]

1.3.2 Extinction cross section calculation

Extinction cross-section spectra (σ_{ext}) of spherical, cubic, and decahedral single metal NPs and clusters were calculated using a 3D electromagnetic solver which applies the finite differential time domain (FDTD) method.[12] For all the calculations, the material for the nanoaprticles was considered to be gold with refractive index from CRC database[13] and the surrounding medium was considered to be water ($n_m = 1.333$) to mimic a colloidal solution of Au NPs. First, the shape and dimensions of the simulated cubic and decahedral NPs were approximated to those of the experimental NPs, observed in TEM micrographs (Fig. S1b, c), with the purpose of calculating σ_{ext} of single Au NPs in good agreement with the corresponding experimental absorbance spectra and to estimate more precisely σ_{ext} of NPs clusters; Blender software was used for NP design.[14] In the case of spherical NPs, Mie theory was applied, and the resulted spectra were also compared to the experimental ones[15] (the best calculated fits are showed in Fig. S3). Good agreement between experimental and theoretical spectra was reached.

Then, σ_{ext} of the single NPs of different sizes and NPs clusters of different number of NPs and gaps were calculated. A typical simulation set up using FDTD consisted in the Au NP (or cluster) centered in a cubic simulation domain limited with perfect matched layers

(PML, 16 layers) and size of 1500 nm in length (twice the maximum applied wavelength) to ensure good convergence. In order to guarantee numerical convergence of the calculations, the auto shutoff threshold was set to 1×10^{-7}— if the fraction of the power remaining in the simulation is less than this value, it triggers the simulation to end — and the simulation time to 1000 fs. Each final σ_{ext} spectrum resulted from averaging 25 individual spectra for various excitation light polarizations with the aim of simulating the unpolarized incoming light. More specificaly, a single NP was set with different randomized rotational angles (polar and azimuthal angles) in each simulation. In the case of the NPs clusters, the rotational angles and the positions of each nanoparticle were randomized, and the rotational angle of the whole cluster was also randomized for each of the 25 simulations that would conform the averaged spectra. The light wavelength range considered ranges from 400 nm to 750 nm (visible spectral range). The absorption (σ_{abs}) and scattering (σ_{sca}) cross sections (not shown here) were determined by using field monitors surrounding the objects under study, and σ_{ext} was then calculated from the arithmetic addition of the scattering and the absorption cross-sections ($\sigma_{ext} = \sigma_{abs} + \sigma_{sca}$).

1.3.3 Conversion of optical spectra into RGB and HSV color models

It has been shown that the Hue (H) parameter, which originates from the HSV color model, exhibits superior sensitivity to the change of refractive index.[16] Analogically, we selected the Hue parameter to describe quantitatively the change of color of a solution during aggregation. To convert σ_{ext} and the experimental absorbance spectra into RGB and HSV space colors, a methodology described previously in Refs. 16 and 17 was followed. First, the absorbance (for experimental results, Figure S7) and the extinction cross section (for theoretical results, Fig. S10 spectra were transformed into Transmittance through the Beer-Lambert Law ($T(\lambda) = 10^{-A(\lambda)}$, where A($\lambda$) is the absorbance as a function of the wavelength). Then, the Transmittance spectra were converted into the tristimulus values (X,Y, and Z) using the following expressions:

$$X = \frac{\int_{400\text{ nm}}^{750\text{ nm}} CIE\tilde{x}(\lambda) \cdot D_{65}(\lambda) \cdot T(\lambda)\, d\lambda}{N},$$

$$Y = \frac{\int_{400\text{ nm}}^{750\text{ nm}} CIE\tilde{y}(\lambda) \cdot D_{65}(\lambda) \cdot T(\lambda)\, d\lambda}{N},$$

and

$$Z = \frac{\int_{400\text{ nm}}^{750\text{ nm}} CIE\tilde{z}(\lambda) \cdot D_{65}(\lambda) \cdot T(\lambda)\, d\lambda}{N}$$

with

$$N = \int_{400\text{ nm}}^{750\text{ nm}} CIE\tilde{y}(\lambda) \cdot D_{65}(\lambda)\, d\lambda,$$

where $CIE\tilde{x}(\lambda)$, $CIE\tilde{y}(\lambda)$, and $CIE\tilde{z}(\lambda)$ are the color matching functions created by the International Commission on Illumination (CIE)[18] in 1931 to describe the perceived colors in human vision (Figure S4), $D_{65}(\lambda)$ is the CIE *standard illuminant D65*, also defined by CIE, which corresponds to the average midday light in Western Europe (Figure S5). The tristimulus values represent the spectral sensitivity of the three cone cell photoreceptors in human eyes. After this, the calculated tristimulus values were transformed to linear RGB by $[R_L G_L B_L] = M\,[XYZ]$, where the transformation matrix is defined by:[19]

$$M = \begin{bmatrix} 3.2406255 & -1.537208 & -0.4986286 \\ -0.9689307 & 1.8757561 & 0.0415175 \\ 0.0557101 & -0.2040211 & 1.0569959 \end{bmatrix}.$$

To optimize the brightness of the color to the perception of human eyes, the calculated RGB values were gamma corrected[16,20] according with the following conditions: if η_L (η_L $=R_L$, G_L, or B_L) is less than or equal to 0.0031308 then $\eta = 12.92\eta_L$, otherwise $\eta = 1.055\eta_L^{(1/2.4)} - 0.055$, where η stands for R, G, or B.

Finally, HSV color model was calculated from the RGB values by using:

$$V = max(R, G, B),$$

$$S = \begin{cases} 0, & \text{if } V = 0 \\ C/V, & \text{otherwise} \end{cases}$$

where $C = V - min(R, G, B)$, and

$$H = \begin{cases} H_S + 1, & \text{if } H_S < 0 \\ H_S, & \text{otherwise} \end{cases}$$

where

$$H_S = \begin{cases} 0, & \text{if } C = 0 \\ \left(\frac{G-B}{C} + 0\right)/6, & \text{if } V = R \\ \left(\frac{B-R}{C} + 2\right)/6, & \text{if } V = G \\ \left(\frac{R-G}{C} + 4\right)/6, & \text{if } V = B. \end{cases}$$

Calculated Hue values were rounded to the nearest hundredth in order to facilitate their interpretation.

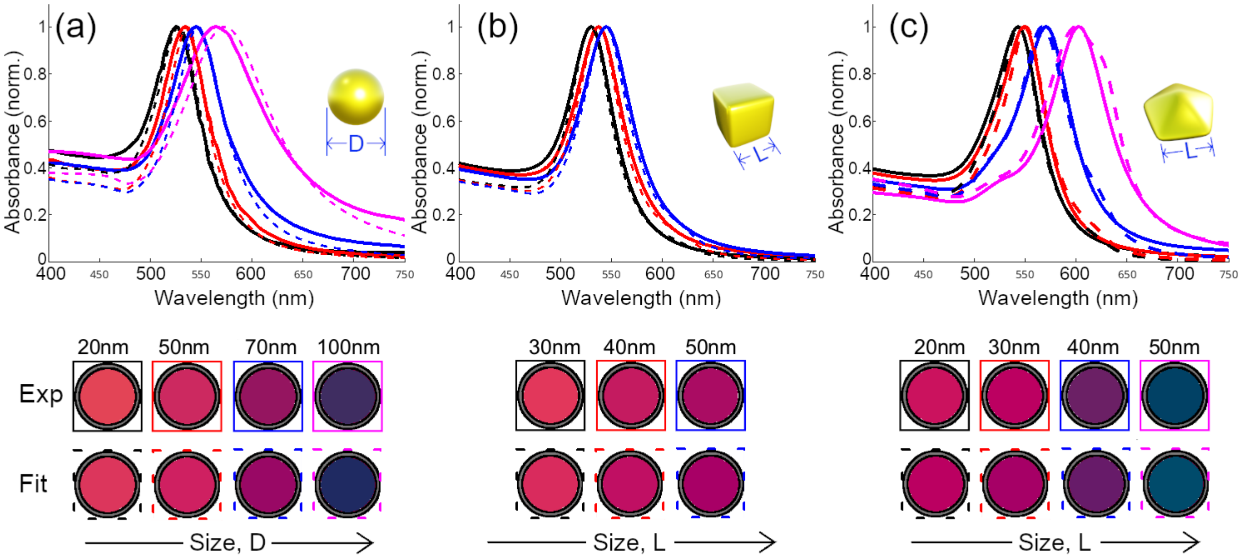

Figure S3: Experimental absorbance spectra (continuous lines) and fitted curves (dashed lines) of single NPs with (a) spheric, (b) cubic, and (c) decahedral shapes and different sizes. In the bottom panel, the RGB colors of each spectrum are shown. The method of calculation is described in the Theoretical Method section.

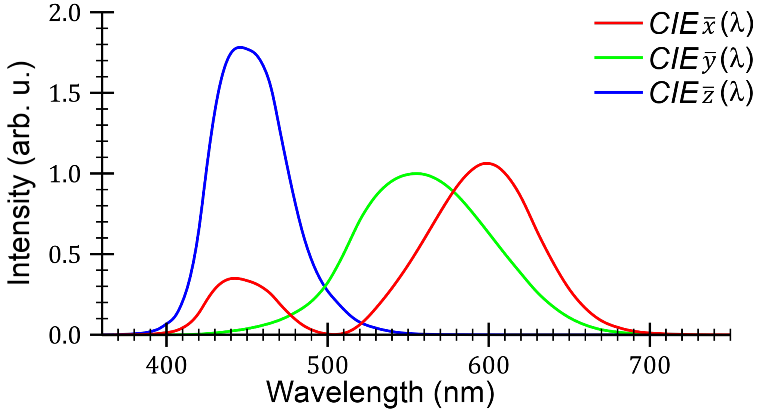

Figure S4: X, Y, and Z CIE-1931 color matching functions ($CIE\tilde{x}(\lambda)$, $CIE\tilde{y}(\lambda)$, and $CIE\tilde{z}(\lambda)$, respectively).

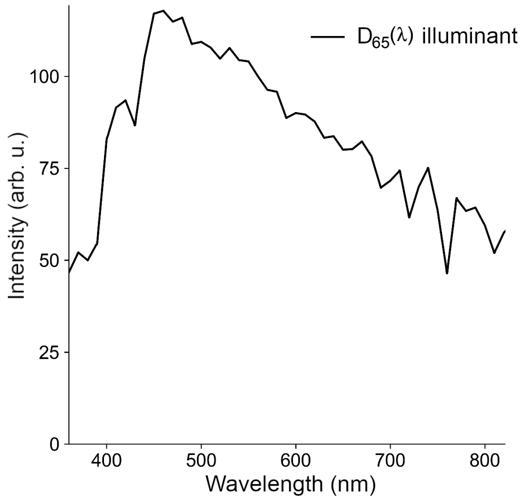

Figure S5: Average midday light irradiance in Western Europe, known as CIE standard illuminant $D65(\lambda)$.

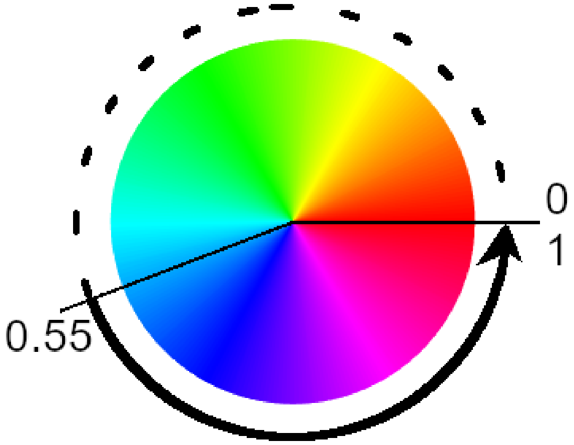

Figure S6: Hue wheel. The range of Hue values used in this study is indicated with the arrow, from 0.55 to 1. The lower limit of Hue value (0.55) was obtained by rounding the minimum value of Hue ($H = 0.5531$) that was obtained for Dec-NPs (experimental).

2 Optical characterization of experimental samples

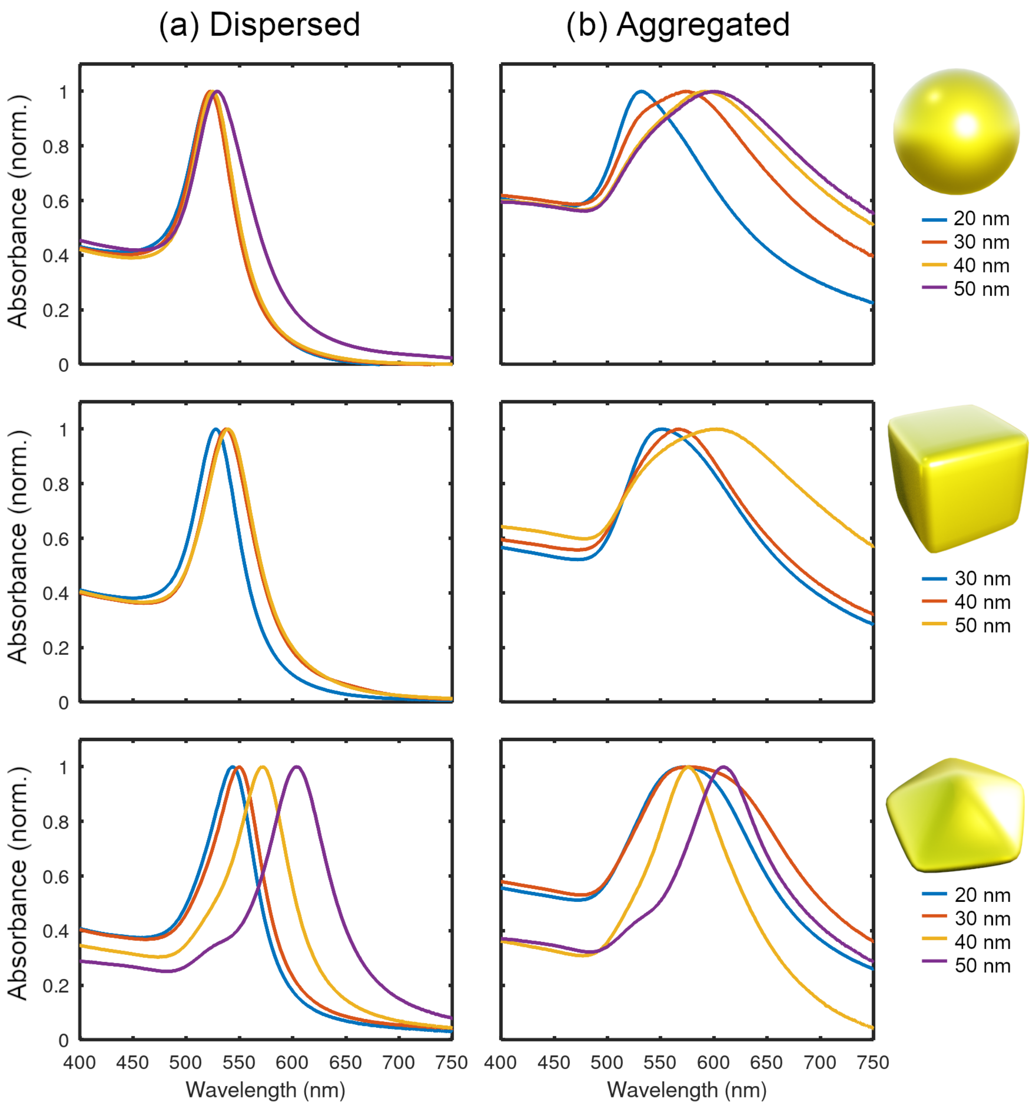

Figure S7: Experimental optical absorbance in the visible spectral range of (a) well-dispersed colloids and (b) aggregated-NPs colloids composed by spherical, cubic, and decahedral gold nanoparticles with different sizes and functionalized with DNA.

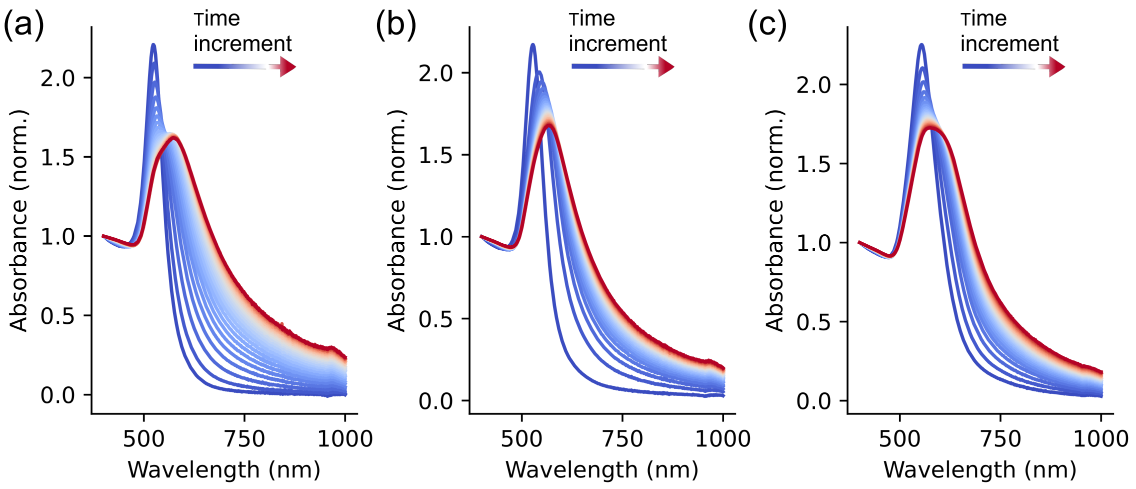

Figure S8: Aggregation kinetics of (a) spherical, (b) cubic and (c) decahedral nanoparticles. The clustering of nanoparticles was tracked for one hour.

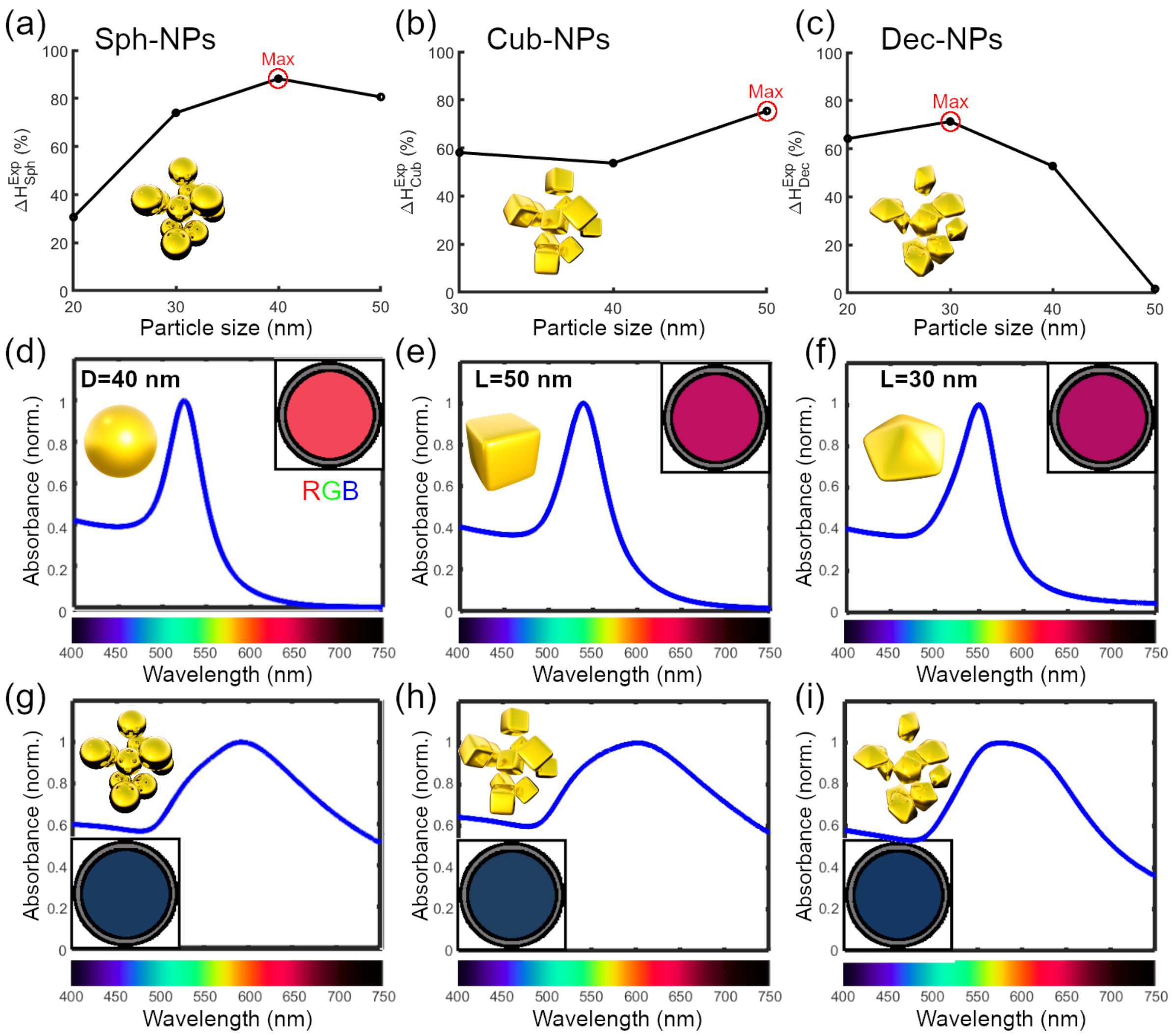

Figure S9: Experimental results. (a-c) Figure of merit ΔH (Eqn. 1 in main text) resulted from the Hue values of the experimental samples (see Figure 4a in main text) of (a) spherical, (b) cubic, and (c) decahedral nanoparticle clusters (ΔH^{Exp}_{Sph}, ΔH^{Exp}_{Cub}, and ΔH^{Exp}_{Dec}, respectively). (d-i) Experimental absorbance spectra and calculated RGB color of (d and g) spherical, (e and h) cubic, and (f and i) decahedral dispersed gold nanoparticles (d-f) and aggregates (g-i), for the sizes in which ΔH is maximum (see red circles in a, b, and c) The bars at the bottom represent the colors perceived by the human eye.[21]

3 Optical characterization of theoretical clusters

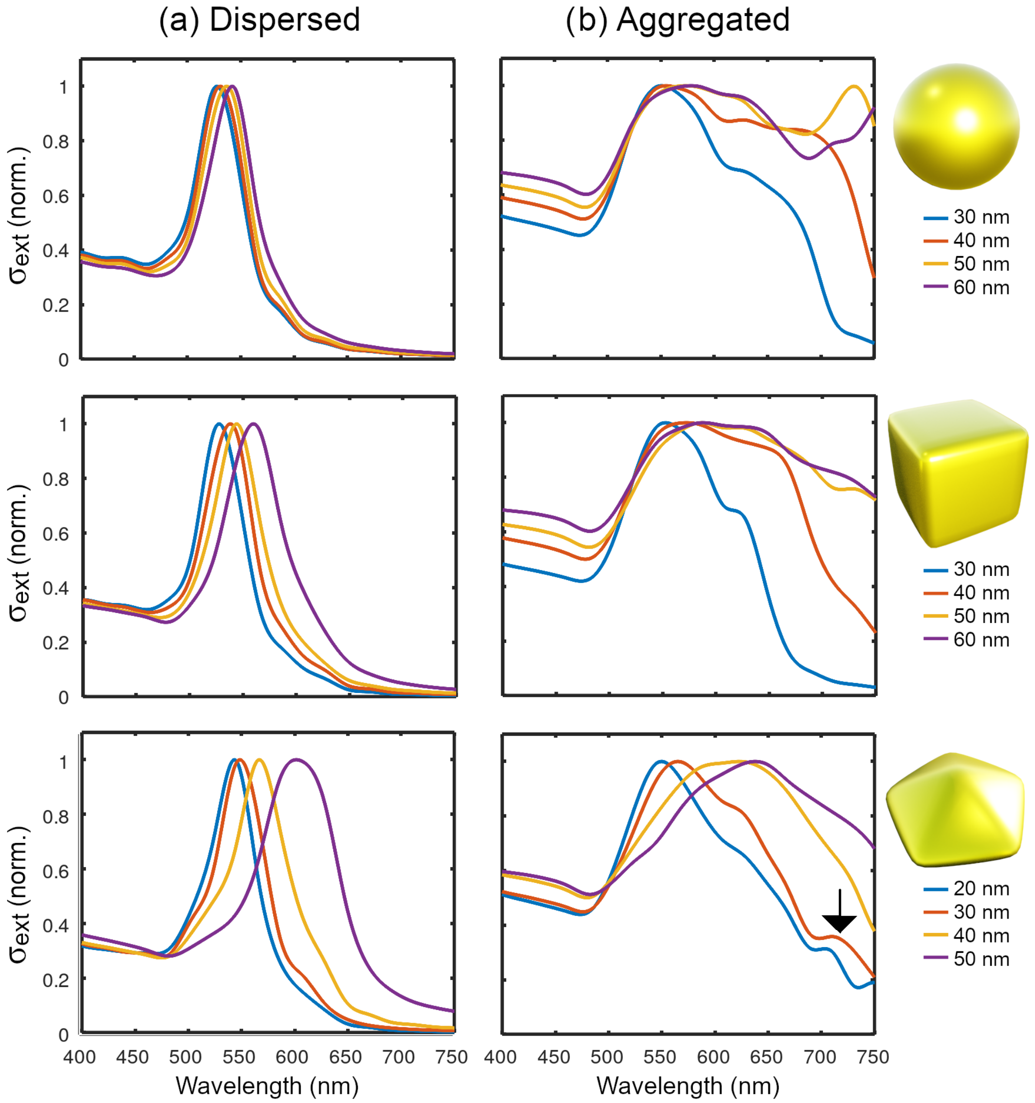

Figure S10: Calculated extinction cross-sections in the visible spectral range of (a) single NPs and (b) NPs clusters formed with 10 NPs and gap distance of 2 nm, for spherical, cubic, and decahedral Au NPs with different sizes

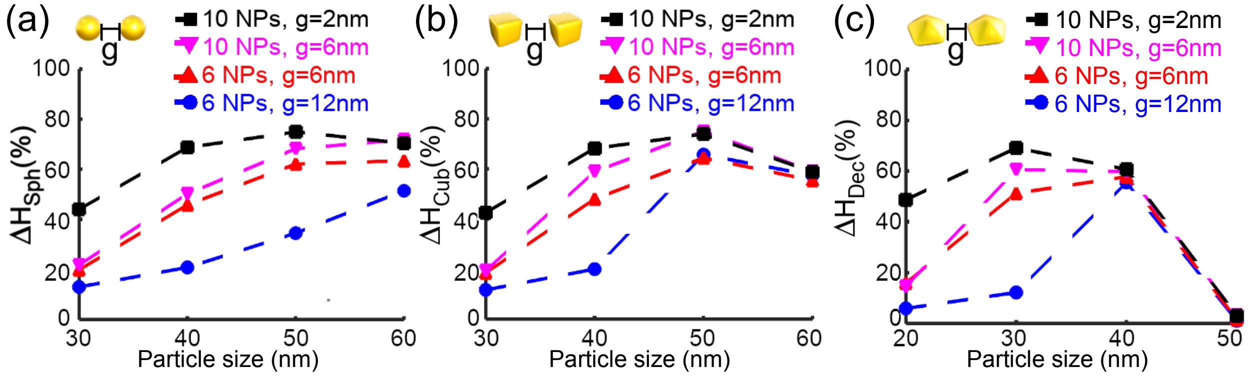

Figure S11: Effect of interparticle gap and NPs per cluster on color transition upon aggregation in model sistems of (a) spherical, (b) cubic, and (c) decahedral nanoparticles. The larger the ΔH, the larger the color difference. Regardless of the number of nanoparticles per cluster (6 or 10), the value of ΔH increases with the decrease of the inter-particle distance. These results indicate that the gap, rather than the number of particles per cluster, establishes color difference upon aggregation.

4 Comparative between ΔE_{76} and ΔH

For the sake of simplicity, we proposed here a quantitative indicator based on the change of Hue (ΔH), while the standardized measure to asses the difference between two colors, ΔE_{76}, is defined as:

$$\Delta E_{76} = \sqrt{(\Delta L)^2 + (\Delta a)^2 + (\Delta b)^2}, \tag{S1}$$

where L, a, and b are the reference stimulus values of the CIE 1976 L*a*b* color space.[22,23] The perception of humans to distinguish between two colors follows the following classification:[23]

- $0 < \Delta E_{76} < 1$: the difference is unnoticeable,
- $1 < \Delta E_{76} < 2$: the difference is only noticed by an experienced observer,
- $2 < \Delta E_{76} < 3.5$: the difference is also noticed by an unexperienced observer,
- $3.5 < \Delta E_{76} < 5$: the difference is clearly noticeable, and
- $5 < \Delta E_{76}$: gives the impression that these are two different colors.

Therefore, to compare our indicator with the standardized measure we calculated ΔE_{76} for numerical and experimental spectra of all cluster configurations (Table S2) — Matlab function: rgb2lab,[24] was used to convert the calculated RGB values to CIE 1976 L*a*b* color space —. We observed that the maxima of ΔE_{76} (see blue values in Table S2) follow the same trend as the values obtained for ΔH in experiments (Figure 5a-c) and simulations (Figure S11). However, it should be stated that the purpose of ΔE_{76} is to determine the value of the lower threshold of differentiation between two colors, that is, the regime where such a difference can be difficult to assess objectively. The analysis of the values in Table S2 shows that virtually all nanoparticles shapes and cluster configurations studied here fall in the range of $3.5 < \Delta E_{76} < 5$, that is, the aggregation of nanoparticles can be clearly differentiated and noticed. Therefore, we postulate that the ΔH indicator is a more convenient measure than

ΔE_{76} for our purpose, since we seek to compare the maxima of color transition rather than assessing qualitatively the lower limits of distinction between two colors.[22]

Table S2: Color difference, ΔE_{76}, between dispersed and aggregated states for both theoretical and experimental (Exp.) optical spectra as a function of nanoparticle size, shape, NPs per cluster, and gap distance (g). The maximum values according to the NP size for each case are highlighted in blue.

**Spherical NPs**					
	**20 nm**	**30 nm**	**40 nm**	**50 nm**	**60 nm**
6 NPs g = 12 nm	—	34	49	56	59
6 NPs g = 6 nm	—	48	68	70	64
10 NPs g = 6 nm	—	51	71	77	73
10 NPs g = 2 nm	—	75	78	75	68
Exp.	70	87	91	75	—

**Cubic NPs**					
	**20 nm**	**30 nm**	**40 nm**	**50 nm**	**60 nm**
6 NPs g = 12 nm	—	30	40	62	44
6 NPs g = 6 nm	—	44	59	61	42
10 NPs g = 6 nm	—	47	65	70	48
10 NPs g = 2 nm	—	66	72	69	48
Exp.	—	78	63	73	—

**Decahedral NPs**					
	**20 nm**	**30 nm**	**40 nm**	**50 nm**	**60 nm**
6 NPs g = 12 nm	19	26	40	3	—
6 NPs g = 6 nm	38	49	43	4	—
10 NPs g = 6 nm	36	58	47	4	—
10 NPs g = 2 nm	58	58	48	4	—
Exp.	68	67	26	4	—

References

(1) Mezzasalma, S. A.; Grzelczak, M.; Sancho-Parramon, J. The crystal field plasmon splitting. *ACS Photonics* **2020**, *7*, 1551–1559.

(2) Sánchez-Iglesias, A.; Winckelmans, N.; Altantzis, T.; Bals, S.; Grzelczak, M.; Liz-Marzán, L. M. High-yield seeded growth of monodisperse pentatwinned gold nanoparticles through thermally induced seed twinning. *J. Am. Chem. Soc.* **2017**, *139*, 107–110.

(3) Mehtala, J. G.; Zemlyanov, D. Y.; Max, J. P.; Kadasala, N.; Zhao, S.; Wei, A. Citrate-stabilized gold nanorods. *Langmuir* **2014**, *30*, 13727–13730.

(4) Hurst, S. J.; Lytton-Jean, A. K. R.; Mirkin, C. A. Maximizing DNA loading on a range of gold nanoparticle sizes. *Anal. Chem.* **2006**, *78*, 8313–8318.

(5) Taylor, R. W.; Esteban, R.; Mahajan, S.; Aizpurua, J.; Baumberg, J. J. Optimizing SERS from gold nanoparticle clusters: Addressing the near field by an embedded chain plasmon model. *J. Phys. Chem. C* **2016**, *120*, 10512–10522.

(6) Moitra, P.; Alafeef, M.; Dighe, K.; Frieman, M. B.; Pan, D. Selective naked-eye detection of SARS-CoV-2 mediated by N gene targeted antisense oligonucleotide capped plasmonic nanoparticles. *ACS Nano* **2020**, *14*, 7617–7627.

(7) Carl, N.; Prévost, S.; Fitzgerald, J. P. S.; Karg, M. Salt-induced cluster formation of gold nanoparticles followed by stopped-flow SAXS, DLS and extinction spectroscopy. *Phys. Chem. Chem. Phys.* **2017**, *19*, 16348–16357.

(8) Esteban, R.; Taylor, R. W.; Baumberg, J. J.; Aizpurua, J. How chain plasmons govern the optical response in strongly interacting self-assembled metallic clusters of nanoparticles. *Langmuir* **2012**, *28*, 8881–8890.

(9) Kruse, J.; Sanromán-Iglesias, M.; Marauri, A.; Rivilla, I.; Grzelczak, M. Coupling Re-

versible Clustering of DNA-Coated Gold Nanoparticles with Chemothermal Cycloaddition Reaction. *ChemSystemsChem* **2023**, *5*, e202200031.

(10) Ghosh, S. K.; Pal, T. Interparticle coupling effect on the surface plasmon resonance of gold nanoparticles: From theory to applications. *Chem. Rev.* **2007**, *107*, 4797–4862.

(11) Park, S. Y.; Lee, J.-S.; Georganopoulou, D.; Mirkin, C. A.; Schatz, G. C. Structures of DNA-linked nanoparticle aggregates. *J. Phys. Chem. B* **2006**, *110*, 12673–12681.

(12) Lumerical Inc. `https://www.lumerical.com/`, Accessed: 2022-01-01.

(13) Weaver, J. H.; Frederikse, H. P. R. In *CRC Handbook of Chemistry*; Lide, D. R., Ed.; CRC Press: Boca Raton, FL, 2005; pp 12–137.

(14) Community, B. O. Blender - a 3D modelling and rendering package. Blender Foundation: Stichting Blender Foundation, Amsterdam, 2018.

(15) Zheng, Y.; Zhong, X.; Li, Z.; Xia, Y. Successive, seed-mediated growth for the synthesis of single-crystal gold nanospheres with uniform diameters controlled in the range of 5–150 nm. *Part. Part. Sys. Charact.* **2014**, *31*, 266–273.

(16) Reinhard, I.; Miller, K.; Diepenheim, G.; Cantrell, K.; Hall, W. P. Nanoparticle design rules for colorimetric plasmonic sensors. *ACS Appl. Nano Mater.* **2020**, *3*, 4342–4350.

(17) Marcus, R. T. In *Color for science, art and technology*; Nassau, K., Ed.; Elsevier Science B.V.: Greenwich, CT, USA, 1998; pp 31–96.

(18) International Comission on Illumination. `https://cie.co.at/`, Accessed: 2022-01-01.

(19) International Color Consortium. `https://color.org/srgb04.xalter`, Accessed: 2022-01-01.

(20) McREYNOLDS, T.; BLYTHE, D. In *Advanced Graphics Programming Using OpenGL*; McREYNOLDS, T., BLYTHE, D., Eds.; The Morgan Kaufmann Series in Computer Graphics; Morgan Kaufmann: San Francisco, 2005; pp 35–56.

(21) Mather, J. Spectral and XYZ Color Functions. 2023; `https://www.mathworks.com/matlabcentral/fileexchange/7021-spectral-and-xyz-color-functions`.

(22) González-Alcalde, A. K.; Reyes-Coronado, A. Large angle-independent structural colors based on all-dielectric random metasurfaces. *Opt. Commun.* **2020**, *475*, 126289.

(23) Mokrzycki, W. S.; Tatol, M. Colour Difference ΔE - a Survey. *MG&V* **2011**, *20*, 383–411.

(24) The MathWorks Inc., 2023; `https://es.mathworks.com/help/images/ref/rgb2lab.html`.
